# Parents’ perspective on children’s access to healthcare services and associated factors: a cross-sectional study

**DOI:** 10.1186/s12889-025-23230-0

**Published:** 2025-07-02

**Authors:** Nona Salimi, Javad Javan-Noughabi, Vahid Ghavami, Reza Matinfar, Fatemeh Kokabisaghi

**Affiliations:** 1 Department of Health Economics and Management Sciences, School of Health, Mashhad University of Medical Sciences, Mashhad, Iran; 2 Social Determinants of Health Research Center, Mashhad University of Medical Sciences, Mashhad, Iran; 3 Department of Biostatistics, School of Health, Mashhad University of Medical Sciences, Mashhad, Iran

**Keywords:** Children, Right to health, Access to health services, Physical access, Quality, Information accessibility

## Abstract

**Background:**

Poor access to health services endangers children’s health and development. Parents’ perspectives are essential for understanding barriers to children’s healthcare access, as they often serve as primary caregivers and decision-makers. This research aimed to explore parents’ perspective on children’s access to healthcare services and its associated factors.

**Methods:**

In this cross-sectional study, 317 parents of children aged ≤ 18, chosen by two-stage cluster sampling participated. Data was collected by the standard questionnaire of the European Patient Forum with some changes. It evaluates overall access to healthcare and its five main dimensions: information availability, affordability, physical accessibility, appropriateness and adequacy. Translation of the questionnaire into Persian included forward and backward translations and expert committee discussions. Cronbach's alpha was 0.935 approving questionnaire’s reliability. A 5-point Likert scale was used to rate the status of access (very low = 1, very high = 5). Descriptive statistics and ordinal logistic regression were used to assess the factors associated with access. Data analysis was performed in STATA17 at a significant level of *p* < 0.05.

**Results:**

Most study participants were women (*n* = 189, 60%) 31–40 years old (*n* = 132, 42%), had a high school degree (*n* = 198, 60.7%), 2–3 children (*n* = 158, 49.8%), average income (*n* = 126, 39.7%) and basic health insurance (*n* = 222,7%). The mean score of children’s overall access to health services was 2.8 ± 1.2, and for its dimensions from highest to the lowest was physical accessibility: 3.19 ± 0.87, appropriateness: 2.81 ± 1.1, information availability: 2.76 ± 0.98, adequacy: 2.60 ± 1.07 and affordability: 1.36 ± 1.07. There was a significant relationship parents’ education level, income and supplementary health insurance and access to healthcare services (*p* < 0.05).

**Conclusions:**

From parents’ perspective, the access of children and it’s all dimensions: information availability, affordability, physical accessibility, appropriateness and adequacy need improvement. Policymakers should ensure all children receive necessary care by providing affordable subsidized health services. Children should have access to age-friendly health information and be supported to participate in healthcare decisions. To make health services more appropriate, it is necessary to train the staff about professional ethics and equal treatment of all patients.

## Background

According to international and regional human rights treaties, all children have the right to enjoy the highest attainable standard of health. The right to health includes the right to timely access to prevention, treatment, and rehabilitation services, as well as determinants of health, including safe water, nutritious food, shelter, and a healthy environment. Health services should be available to all children without discrimination of any kind. These services should be acceptable to society and of satisfactory quality. Children need special protection to enjoy their rights. Governments should use all the budgetary, legal, administrative, judicial and promotional means to promote children rights [1].

Universally, every year, about ten million children under five die due to poor access to healthcare services [2]. In different countries, children face diverse challenges to access health services. Strong predictors for access to quality healthcare in the USA included health insurance, higher income, and available primary healthcare networks [3]. A review of the use of health services by children in Brazil showed that three types of factors, including the ones related to children (age and health status), caregivers (education and health literacy), and healthcare providers (availability), influenced the use of services [4].

Article 29 of Iran’s Constitution recognizes everyone's right to medical care and obligates the government to use public revenues to improve the health system and people’s standard of living. In the law on the Structure of the Comprehensive Welfare and Social Security System 2002, social security (including health insurance and rehabilitation) was defined as everyone's right. Moreover, vulnerable groups, including indigent families, legal immigrants, and children living in institutions, receive subsidized health insurance and care. To provide primary healthcare to everyone, the healthcare network has been established in the rural and urban areas of Iran. Many health services, including family and child health, prevention and control of diseases and vaccinations are provided in primary healthcare facilities free of charge. For secondary and tertiary health services, children may be presented to public and private hospitals and clinics that are not affordable for everyone. Having health insurance is neither obligatory, nor free. Parents register their children with an insurance organization and pay the premium. Out-of-pocket payments for healthcare are more than 55% in Iran. Despite the significant growth of children's health indicators at the national level, disparities exist in access to medical services among different socio-economic groups. In remote and disadvantaged regions, the rates of child mortality, low birth weight, and malnutrition are higher than the national average, and some children do not have access to adequate nutritious food, proper shelter, and healthcare facilities [5]. Studies show that the distribution of health sector resources between different regions is not equal. The situation is favorable in megacities such as Tehran, but some other cities are underprivileged [6]. A study conducted in the city of Tabriz, Iran indicated the unfair distribution of health services and unequal access in different urban areas [7].

Access to health services is beyond the availability of services and access barriers might be related to knowledge or culture of the population. Four factors; personal, geographic, financial, health centers related, impact people's access to health services. A study showed that financial and geographical barriers decreased the access to healthcare services in Share-ray City, Iran. Among the geographic variables, the location of the service centers and the availability of transportation means, and among the financial factors, the service tariff rate, had significant effects on people's access to health services [8]. Moreover, health services use varies among different groups of children and is influenced by various factors. The most important reasons for children’s low utilization of health services in Qom City, Iran, from the health service providers’ point of view were inadequate health literacy and information about available services, and access to quality services [9]. A study found that from parents'perspective, the most important barrier to dental care was the high costs in Kerman, Iran [10]. Insufficient dental insurance coverage, long waiting times and high costs were barriers to accessing dental care for children in Tehran, Iran [11]. The study on the perceptions of autistic children’s parents about access to rehabilitation services in Iran found the lack of a support system and comprehensive diagnostic and rehabilitation guidelines as access barriers [12]. A study in Kermanshah City, Iran, showed that the physical access of children and adolescents to hospitals and emergency centers was acceptable [13].

Though the overall access of the population to health services has been studied before, little is known about children’s access to health services in Iran. Parents’ perspectives are essential for understanding barriers to healthcare access for children, since they often serve as primary caregivers and decision-makers and children rely on them to meet their needs. This study aimed to understand parents’ perspective on children’s access to healthcare services and its associated factors.

## Methods

A descriptive-analytical study with a cross-sectional design was conducted to investigate parents'perspectives on their children’s access to health services and the associated factors it in the City of Mashhad in northeast Iran in 2023. Mashhad is the second most populous city in the country, with a population of more than 3 million people. It receives about 30 million domestic and foreign tourists annually.

STROBE reporting guideline [14] for cross-sectional studies was used in this study.

### Study population and sample

According to the National statistics Center in Mashhad, the population aged younger than 18 was 981.238 in 2019 [15]. The study population consisted of parents who visited Primary Health Care (PHC) facilities in different areas of the city. We used the Formula 1, to estimate sample size in this study. Since there was not any information about children’s access to health services in Mashhad in previous studies, we considered a small Cohen effect size (d) of 0.2 [16]. With a type one error of 0.05, a power of 90% and anticipated attrition rate of 15%, the estimated sample size was 317.

Formula 1) sample size estimation.

$$n=\frac{\left(Z_{1-{\frac{a}{2}}} + Z_{1-\beta}\right)^2}{d^2}$$

In this study, a two-stage cluster sampling method was adopted. For this purpose, the city was divided into five areas. Three areas were chosen randomly, and five PHC facilities were randomly selected from each area. Then, by convenient sampling, 21 parents were selected from each facility to participate in the study. The inclusion criteria included having children younger than 18 and living in Mashhad.

### Data collection tool

In this study, the standard questionnaire of the European Patients Forum about access to medical services was used. This questionnaire examines the access to health services in 5 dimensions, including information availability, affordability, physical accessibility, appropriateness and adequacy [17]. Each dimension has a main question and some sub-questions: information availability (two sub-questions), affordability (six sub-questions), physical accessibility (four sub-questions), adequacy of health services (10 sub-questions on communication and quality of care) and appropriateness (two sub-questions about good behaviour and non-discrimination). In addition, the questionnaire included nine questions related to socio-demographic information of study participants such as age, gender, education, number of children, having a child with a chronic condition, working status, income, health insurance, and supplementary health insurance.

The original version of the questionnaire is in English. The translation into Persian included forward and backward translations and expert committee discussions. Five experts in the health sector qualitatively assessed the questionnaire's content validity. The ambiguous items were corrected and changes were made to make the questionnaire appropriate for evaluating parents’ perspective on children’s access. The internal consistency of the questionnaire was evaluated by a test–retest method with an interval of two weeks with 30 people from the target population. The value of Cronbach's alpha was 0.935, which is an acceptable value and shows the reliability of the tool.

### Data collection and analysis

The questionnaires were self-administered and delivered in paper and pen format. Data was collected between October 2022 and March 2023. One of the researchers attended the health centers and explained the purpose of the study and how to fill out the questionnaire to all participants. He distributed and collected the questionnaires, and whenever needed, provided more information to the participants.

The demographic and access data were subjected to descriptive analysis. A 5-point Likert scale was used to score overall access and main dimensions of children's access to health services (very low, low, moderate, high and very high = 1–5). Mean scores were calculated by averaging individual responses within each access dimension. The sub questions providing more details were answered by Yes/No and I did not need it/I do not know options or Never, Rarely, Sometimes, Most of the time and Always.

The factors influencing children's overall access to health services were investigated using ordinal logit regression. Access was the dependent variable, and age, gender, education, the number of children, having a child with a chronic condition, working status, income, health insurance, and supplementary health insurance were the independent variables. Data analysis was done in STATA version 17 software at a significant level of p ≤ 0.05.

### Findings

1. Sample characteristics

132 study participants (42%) were in the age group of 31–40 years, and about 60% (*n* = 189) were women. All participants were literate, and 198 parents (60.7%) had a high school degree. Most participants (49.8%) had 2 or 3 children (*n* = 158). 2.1% of participants had a child with a chronic disease (*n* = 15). In 63.7% of families, one parent worked (*n* = 202). 39.7% of participants declared their income level is average (*n* = 126). 70% of children had basic health insurance (*n* = 222), and 36% had supplementary insurance (*n* = 115) (Table 1).

**Table 1:** Demographic information of study participants

Variable	Categories	Number	Percentage
Age	≤ 20	25	7.9
	21–30	81	25.6
	31–40	132	41.6
	41–50	58	18.3
	50 ≥	21	6.6
Gender	Woman	189	59.6
	Man	128	40.4
Education	Illiterate	0	0
	Primary school	6	1.8
	High school	198	60.7
	University education	119	37.5
Number of children	One	117	36.9
	02-Mar	158	49.8
	More than 3	42	13.2
A child with a chronic condition	Yes	15	2.1
	No	302	97.9
Working	Both parents	64	20.2
	One of the parents	202	63.7
	None of the parents	51	16.1
Income (participants’ perception)	Very low	78	24.6
	Low	82	25.9
	Average	126	39.7
	High	31	9.8
Health insurance	Yes	222	70
	No	95	30
Supplementary health insurance	Yes	115	36.3
	No	202	63.7
Total		317	100

2) Children’s access to health services

The mean score of children’s overall access to health services was 2.8 ± 1.2, and for its dimensions from highest to the lowest was physical accessibility: 3.19 ± 0.87, appropriateness: 2.81 ± 1.1, information availability: 2.76 ± 0.98, adequacy: 2.60 ± 1.07 and affordability: 1.36 ± 1.07 (Table 2).

**Table 2:** Score of overall access to health services and its dimensions

Dimension	Responses N (%)					Mean ± SD	Total number of participants
	Very low	Low	Moderate	High	Very high		
Information Availability	46(14.5)	56(17.6)	151(47.6)	53(16.7)	11(3.5)	2.76 ± 0.98	317
Affordability	53(16.7)	83(26.1)	141(44.5)	29(9.1)	11(3.5)	1.36 ± 1.07	317
Physical Accessibility	16(5.06)	23(7.27)	172(54.4)	62(19.6)	43(13.6)	3.19 ± 0.87	316
Adequacy	62(19.5)	75(23.6)	120(37.8)	42(13.5)	17(5.3)	2.60 ± 1.07	316
Appropriateness	20(6.5)	52(16.9)	172(56.0)	56(18.2)	7(2.2)	2.81 ± 1.1	307
Overall access	53(16.7)	51(16.0)	133(41.9)	61(19.2)	19(5.9)	2.8 ± 1.2	317

43.8% (*n* = 139) of parents declared that they had easy access to the information of available health facilities, and 56.5% (*n* = 179) believed that the information was understandable and useful. However, 50.8% (*n* = 161) considered the information on medical costs was not transparent (Table 3).

**Table 3:** Access to the information of available health facilities

Dimensions of information availability	Frequency (percentage)		
	I do not know	No	Yes
Easy access	93 (29.4)	85 (26.8)	139 (43.8)
Being understandable	72 (22.7)	66 (20.8)	179 (56.5)
Usefulness	82 (25.8)	70 (22.1)	165 (52.1)
Transparency in healthcare costs	52 (16.4)	161 (50.8)	104 (32.8)
Number of participants	317 (100%)		

More than 20% (*n* = 67) of parents had high or very high financial problems in paying their child's medical expenses. While 55% (*n* = 168) of respondents declared low and very low financial problems. More than 60% (*n* = 167) of participants believed that health insurance coverage for children's medical needs was low or very low. More than 70% (*n* = 237) of participants had a low or very low ability to pay for dental care and corrective surgeries. 48% (*n* = 153) of parents have had to reduce life’s necessities to meet the medical needs of their children. Also, about 70% (*n* = 209) of parents did not have enough resources to buy supplementary insurance for their children. About 47% (*n* = 149) of parents delayed their child's treatment at least once due to the inability to pay medical costs last year (Table 4).

**Table 4:** Affordability of health services and health insurance

Number of participants	Frequency (percentage)					Questions	
	Very low	Low	Moderate	High	Very high		
243	73 (23)	95 (30)	82 (26)	35 (11)	32 (10)	Financial problems in paying the child's medical expenses	
317	129 (40.7)	75 (23.7)	76 (24)	29 (9.1)	8 (2.5)	The coverage of the child's medical expenses by insurance	
277	26 (8.2)	46 (14.6)	139 (43.8)	59 (18.6)	47 (14.8)	General practitioners	Affordability of specific health services
317	82 (25.9)	61 (19.3)	106 (33.4)	39 (12.3)	29 (9.1)	Medical specialists	
297	108 (34)	81 (25.6)	85 (26.8)	29 (9.2)	14 (4.4)	Therapists	
317	72 (22.6)	69 (21.8)	121 (38.2)	36 (11.4)	19 (6)	Hospital care	
227	84 (26.5)	85 (26.8)	109 (34.4)	25 (7.9)	14 (4.4)	Diagnosis and medical equipment	
307	155 (48.9)	82 (25.9)	36 (11.4)	28 (8.8)	16 (5)	Dental care	
317	163 (51.3)	68 (21.5)	57 (18)	19 (6)	10 (3.2)	Corrective surgeries	
	Yes	No	Supplementary aspects				
279	153 (48.3)	164 (51.7)	Reducing the expenses of living necessities to pay for the child's treatment				
227	98 (30.9)	219 (69.1)	Having enough money to buy supplementary insurance for the child				
317	148 (47)	169 (53)	Delay in treatment because of the inability to pay the expenses				

According to study participants, children had fair physical access to most health services. Delays mostly happened in accessing medical specialists and medical laboratories (Table 5).

**Table 5:** Delays in accessing services due to the distance from service centres

Dimensions	Frequency (percentage)			No. participants
	Yes	No	child did not need it	
Pharmacy	36 (11.4)	210 (66.2)	71 (22.4)	317
Hospital care	47 (14.8)	112 (35.3)	152 (49.9)	311
Medical equipment	61 (19.2)	111 (35)	143 (45.8)	315
Medical laboratories	84 (26.5)	134 (42.3)	99 (31.2)	317
General practitioners	52 (16.4)	192 (60.9)	72 (22.7)	316
Medical specialists	114 (36)	133 (42)	70 (22)	317

Adequacy of health services is related to multiple factors such as the participation of the child and parents in the decision-making process, and quality of services. As shown in Table 6, more than 30 percent (*n* = 100) of the respondents believed that they and their children were either never or rarely involved in making decisions about child’s treatment. Also, more than 40% (*n* = 135) of parents declared that the children rarely or never received information from their doctors.

**Table 6:** Adequacy of health services for children

Dimensions	Frequency (percentage)					overall
	Always	Most of the time	Some times	Rarely	Never	N (%)
Effective communication with healthcare providers	44 (13.9)	74 (23.3)	99 (31.2)	67 (21.1)	33 (10.4)	317 (100)
Receiving enough information from the doctor	22 (6.9)	62 (19.6)	98 (30.9)	77 (24.3)	58 (18.3)	317 (100)
Participation in decision-making	32 (10.1)	74 (23.3)	111 (35)	58 (18.3)	42 (13.2)	317 (100)
Receiving information about the safety of the child's treatment	27 (8.5)	89 (28.1)	91 (28.7)	71 (22.4)	39 (12.3)	317 (100)
Adjusting the treatment according to the child's needs	34 (10.7)	74 (23.3)	124 (391)	50 (15.8)	35 (11)	317 (100)
The attention of personnel to the quality of medical services	28 (8.8)	79 (24.9)	122 (38.5)	61 (19.2)	27 (8.5)	317 (100)
Satisfaction with the safety of medical services	31 (9.8)	80 (25.2)	128 (40.4)	57 (18)	21 (6.6)	317 (100)
Satisfaction with the continuity of services provided over time	30 (9.5)	75 (23.7)	132 (41.6)	55 (17.4)	25 (7.9)	317 (100)

Appropriateness of health services necessitates non-discriminatory behaviour with all. The parents who gave very low and low scores to the appropriateness of health services (*n* = 72, 23.4%) were asked about the type of discriminatory behaviour they experienced. They could choose more than one option. The most common discriminatory behaviour was misbehaviour (30.4%, *n* = 78). The most frequent basis for discriminatory behaviour was low income mentioned 67 times (24.1%) (Table 7).

**Table 7:** Discriminatory behaviour of healthcare providers from parents’ perspective

Dimensions	Frequency	Percentage
Type of discriminatory behaviour^a^		
Denial of the rights	57	22.2
Misbehaviour	78	30.4
Refusal to provide services	42	16.4
Inappropriate language	32	12.5
Others	47	18.3
The basis of discriminatory behaviour^a^		
Being a young parent	31	11.1
Disability of parent or child	11	3.95
Having a child with a chronic illness	13	10.2
The old age of parents	9	4.67
Belonging to a minority group or immigrant family	30	10.7
Being a woman	15	5.39
Being a man	26	9.35
Low Income	67	24.1
Religion	14	5.03
Low education	15	5.39
Others	47	16.9
Number of participants	72	

^a^Multiple options could be selected

3) Bivariate and multivariate analysis

The results of ordinal logistic regression showed a statistically significant positive relationship between having university education and children’s access to medical services so that the chance of more access to health services in households with university education was 2.07 times of other households (*p* = 0.005). Also, a significant positive relationship was found between parents'income and children’s access to medical services. The chance of more access to health services among children of high-income parents was 4.81 times of children of very low-income parents. Moreover, a meaningful relationship existed between children's supplementary health insurance and their access to medical services. Lack of supplementary insurance reduced the chance of accessing medical services by 0.43 compared to the other group (Table 8).

**Table 8:** Relationship between factors affecting children’s overall access to health services

Variable	Categories	Odds Ratio (OR)	*P*-Value
Age of parent	≤ 20	Reference	
	21–30	1.46	0.428
	31–40	1.42	0.94
	41–50	2.18	0.116
	50 ≤	0.77	0.653
Gender	Woman	Reference	
	Man	0.75	0.176
Education	High school diploma and less	Reference	
	University education	2.07	0.005
Number of children	One	Reference	
	02-Mar	0.93	0.761
	More than3	1.38	0.403
A child with a chronic condition	Yes	Reference	
	No	0.58	0.39
Working	Both parents	Reference	
	One of the parents	0.83	0.484
	Not working	0.47	0.051
Income	Very low	Reference	
	Low	2.69	0.002
	Moderate	2.32	0.003
	High	4.81	< 0.001
Health insurance	Yes	Reference	
	No	0.63	0.079
Supplementary health insurance	Yes	Reference	
	No	0.57	0.028

## Discussion

This study investigated parents'perspectives on their children’s access to health services. Access to health services has several dimensions, including information availability, affordability, physical accessibility, appropriateness and adequacy.

The mean score of children’s overall access to health services was 2.8 ± 1.2. One fourth of the studied parents reported that their child's overall access to medical services was satisfactory the last 12 months. In line with our findings, a study on children's health rights in Iran showed that access to medical services in remote and slum areas was low and needed to be improved. In addition, the financial accessibility to healthcare for children belonging to families with low economic and social status was not desirable and required special attention from the government [18]. Dental care, corrective surgeries and therapies were the most expensive care for children in this study. Dental care is not adequately covered by basic health insurance. A found similar findings. They studied the access of children to oral and dental health services in Tehran in 2017 and showed that 94% of parents experienced at least one obstacle to accessing dental services. The most common obstacles were the non-coverage of dental expenses by insurance, long waiting times, and the high treatment costs. Moreover, only half of the parents were satisfied with the services received by their children [11]. Similar studies in Isfahan and Qom Cities, Iran, reported that the main barriers to accessing dental services were high treatment costs, lack of insurance coverage and children’s fear of dentistry [19, 20].

The parents gave a mean score of 2.76 ± 0.98 to information availability. The present study showed that access to healthcare information was satisfactory for one fifth of studied parents. The information on available health facilities was easy to acquire, understandable, and useful according to almost half of the participants, but the lack of transparency in treatment costs was observed. In addition, the respondents were not sure about the access of disabled people to health information. In line with this finding, in a study by the European Patient Forum (2016), the study participants stated that sometimes the health information was not understandable or the transparency about treatment costs was not enough [17]. An Iranian study showed that in recent years, effective measures have been taken to enhance health literacy and access to health information. However, still, teenagers do not have enough access to health information according to their age [18]. Also, a study on the access of young people to health services and socio-geographical distribution of health facilities in Tehran showed that one of the barriers to accessing health services was lack of knowledge on available services, which is in agreement with our findings [21].

The parents gave a mean score of 1.36 ± 1.072 to affordability of services. Almost 12% of studied parents had high and very high financial access to medical services. Similarly, a study in selected public hospitals of Isfahan City, Iran, showed that the exponential increase in healthcare costs caused people to refrain from seeking expensive services, such as medical specialists, surgeries, and medical devices [22]. In an EU-wide study, about half of the patients sometimes faced financial problems accessing health services, and some had to reduce living expenses to cover treatment costs or delayed seeking services [17].

The parents gave a mean score of 3.19 ± 0.87 to physical accessibility. Physical access of children to medical services such as pharmacies, general practitioners, medical specialists, and hospital care was satisfactory for one third of parents. They rarely had to seek health services needed by their children outside the city. Similar to our findings, a study showed that health facilities were not distributed according to the population distribution of Mashhad, Iran. In the peripheral areas of the city, the number of health facilities was not enough. Unequal distribution of medical centers can lead to long waiting times and difficulties in commuting to the centers [23]. To improve child health in Iran, rational distribution of resources and fulfilling rural and underserved region’s needs, educating mothers, improving the health information system, and considering social determinants of health were suggested [24].

The parents gave a mean score of 2.60 ± 1.07 to the adequacy of health services for children. In this study, almost one fifth of parents declared that the adequacy of medical services received by their children was satisfactory. Adequacy includes the quality of care and patient communication and involvement in decision making. More than 30 percent (*n* = 100) of the respondents stated that they and their children were either never or rarely involved in making decisions about child’s treatment. Also, more than 40% (*n* = 135) of parents declared that the children rarely or never received information from their doctors. The study by European Patient’s Forum found that access to and adequacy of healthcare services needed to be improved. Most patients stated that the doctors never asked their opinion about the treatment options and procedures [17]. A study in Urmia, Iran, showed that based on the opinions of the service recipients, the factors affecting the quality of medical services in emergency departments included the behaviour, skills and efficiency of employees, the length of the service delivery processes, the guidance and direction provided to the patients, and respecting their dignity [25].

Appropriateness of health services necessitates non-discriminatory behavior with all. The parents gave a mean score of 2.81 ± 1.1 to health services appropriateness. In the present study, one fifth of parents (*n* = 63) stated that the services provided to their children was appropriate which means they were treated fairly and without discrimination based on age, gender, disability, chronic illness, mental diseases, belonging to a minority group or immigrant family, income level, religion, education level and number of children. Among the parents who gave very low and low scores to the appropriateness of health services (*n* = 72, 23.4%), the most common discriminatory behavior was misbehavior (30.4%, *n* = 78) and the most frequent basis for discriminatory behavior was low income mentioned 67 times (21.4%). Non-discrimination in health services has been studied at the European level. Similar to our findings, in the study by European Patients'Forum, some patients experienced stigma and discrimination. It mostly happened to people with chronic diseases [17].

There was a significant relationship between parents’ education level, income and supplementary health insurance, and access to medical services (*p* < 0.05). In line with our results, a study on the socio-economic factors determining households’ utilization of health services in Tehran showed a significant relationship between households’ income, the education level of the household’s head, having a person with a chronic disease in the family, and health services conditions. Moreover, having health insurance was an effective factor in accessing healthcare services [26].

### Limitations of the study

This study provides useful information about different aspects of children’s access to health services. The results can help policymakers fulfil their obligations regarding children. However, the parental perspective might differ from the children’s actual access experience or from healthcare providers’ perspectives. Moreover, similar to other studies with a cross-sectional design, this study cannot establish a cause-and-effect relationship. In addition, self-reporting by parents may introduce reporting bias.

## Conclusion

From parents’ perspective, the access of children and it’s all dimensions: information availability, affordability, physical accessibility, appropriateness and adequacy need improvement. The situation of physical accessibility was the best and the affordability the worst and challenging. All disadvantaged children, particularly the ones with low socio-economic conditions, should receive governmental support to overcome the access barriers. The government should provide free or subsidized health services and insurance to indigent families. Children should have timely access to prevention, treatment, and rehabilitation services. Childhood is the best time to promote health literacy; children should receive health information appropriate to their age. They should have the opportunity to participate in making decisions about their health and treatment options. Establishing adolescent-friendly health centres is a must. There should be a comprehensive plan for children's health and welfare involving all related bodies.

Nowadays, Iran’s economic situation is a real challenge and barrier to enhance access to care. To protect vulnerable population of children, the adoption of relatively low-cost targeted programs would be helpful. By reforming health system processes and establishing the referral system, better use of resources and increasing access will be possible. Future studies are needed to address the situation of different disadvantaged children and their access to health services. Moreover, longitudinal studies to explore causality and qualitative studies to gain deeper insights into access barriers are necessary.

## Data Availability

Data is provided within the manuscript or supplementary information files.
